# Severe Bloodstream Infection due to KPC-Producer *E coli* in a Renal Transplant Recipient Treated With the Double-Carbapenem Regimen and Analysis of In Vitro Synergy Testing: A Case Report

**DOI:** 10.1097/MD.0000000000002243

**Published:** 2016-02-18

**Authors:** Alessandra Oliva, Alessia Cipolla, Francesca Gizzi, Alessandra D’Abramo, Marco Favaro, Massimiliano De Angelis, Giancarlo Ferretti, Gianluca Russo, Marco Iannetta, Claudio M. Mastroianni, Maria T. Mascellino, Vincenzo Vullo

**Affiliations:** From the Department of Public Health and Infectious Diseases, Sapienza University of Rome (AO, AC, FG, AD, MDA, GF, GR, MI, CMM, MTM, VV) and Department of Experimental Medicine and Biochemical Sciences, University of Rome Tor Vergata (MF), Rome, Italy.

## Abstract

Transplant recipients are at high risk of infections caused by multidrug resistant microorganisms. Due to the limited therapeutic options, innovative antimicrobial combinations against carbapenem-resistant *Enterobacteriaceae* causing severe infections are necessary.

A 61-year-old woman with a history of congenital solitary kidney underwent renal transplantation. The postoperative course was complicated by nosocomial pneumonia due to *Stenotrophomonas maltophilia* and pan-sensitive *Escherichia coli*, successfully treated with antimicrobial therapy. On postoperative day 22, diagnosis of surgical site infection and nosocomial pneumonia with concomitant bacteremia due to a *Klebisella pneumoniae* carbapenemase-producer *E coli* was made. The patient was treated with the double-carbapenem regimen (high dose of meropenem plus ertapenem) and a potent synergistic and bactericidal activity of this un-conventional therapeutic strategy was observed in vitro. Despite a microbiological response with prompt negativity of blood cultures, the patient faced a worse outcome because of severe hemorrhagic shock.

The double-carbapenem regimen might be considered as a rescue therapy in those subjects, including transplant recipients, in whom previous antimicrobial combinations failed or when colistin use might be discouraged. Performing in vitro synergy testing should be strongly encouraged in cases of infections caused by pan-drug resistant strains, especially in high-risk patients.

## INTRODUCTION

The emergence and global spread of infections caused by carbapenem-resistant *Enterobacteriaceae* (CRE) are of great concern worldwide because they are associated with high mortality rates.^1,2^

Resistance to carbapenems is mainly mediated by 2 mechanisms: production of extended spectrum beta-lactamases combined with porin loss or production of carbapenem-hydrolyzing enzymes, namely carbapenemases.^3^

In the presence of carbapenemases, carbapenems at standard dosage might be ineffective; thus, polymixin-based antimicrobial combinations have emerged as the milestone of CRE treatment.^4^ However, increasing rates of polymixin resistance have been recently reported.^5^

In this setting, an innovative approach based on double-carbapenem combination (ertapenem [ERT] followed by high dose of meropenem [MEM]) has been shown to be effective against *Klebsiella pneumoniae* carbapenemase (KPC)-producer *K pneumoniae*, which represents the most common CRE reported in the literature.^6–9^

Given extensive healthcare contact before and after transplant and the need for lifelong immunosuppression, transplant patients are vulnerable to several infections, including those caused by multidrug resistant (MDR) organisms.^10^

The risk of infection after transplantation changes over time and is a function of the state of immunosuppression.^11^ Meanwhile, patients waiting for transplantation might become colonized with nosocomial microorganisms and eventually develop systemic infections due to these challenging pathogens early after transplantation.^11^

Herein, we describe a case of bloodstream infection (BSI) caused by a KPC-producer *Escherichia coli* in a renal transplant patient treated with the double-carbapenem regimen.

## CASE REPORT

A 61-year-old woman with a history of congenital solitary kidney underwent hemodialysis because of end-stage renal failure in 2004. In July 2014, she underwent deceased donor renal transplantation. Simultaneously, a double-pigtail stent was placed and an endoarterectomy of the left iliac artery was performed because of severe stenosis. The postoperatory course was complicated by peri-renal lymphocele and bleeding of the left iliac artery. Immuno-suppressive therapy with tacrolimus was started and its serum concentration was regularly measured. On postoperative day 10, the patient developed fever (T 38.5°C), chills, and dyspnea, with pain on palpation in the lower abdominal quadrants. A chest X-ray showed the presence of bilateral pulmonary consolidations and culture of bronchoalveolar lavage (BAL) grew *Stenotrophomonas maltophilia* and a pan-sensitive *E coli*. An abdominal computed tomography scan displayed a prevesical hematoma (9 × 5 × 7 cm) requiring drainage placement. Cytomegalovirus (CMV)-DNA was 44,400 copies/mL (detection limit <200 copies/mL). According to creatinine clearance (37 mL/min), a postantibiogram antimicrobial therapy consisting of MEM 1 g every 12 h, trimethoprim/sulfamethoxazole (TMP/SMX) 320/1600 mg divided every 8 h and levofloxacin 750 mg every 48 h was started, with clinical (defervescence) and radiological (disappearance of pulmonary consolidations) responses. Furthermore, intravenous therapy with ganciclovir 150 mg every 24 h was started, with a prompt undetectability of CMV viremia.

**FIGURE 1:**
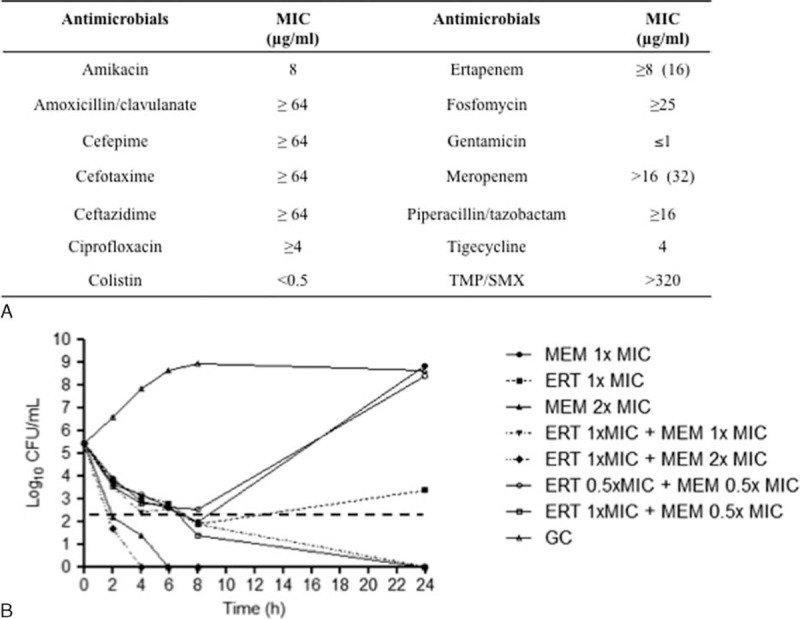
(A) MICs distribution of KPC *Escherichia coli* by VITEK-2 system. Values in brackets refer to MIC determination by broth macrodilution method (BMD). (B) Time–kill studies for ertapenem, meropenem, and ertapenem plus meropenem against KPC *E coli*. The horizontal line represents a reduction of 3 log10 CFU/mL compared with the initial bacterial count. Bactericidal activity was defined as a ≥3-log10 CFU/mL reduction of the initial bacterial count at each time point whereas synergy was defined as a ≥2-log10 decrease in CFU/mL between the combinations and its most active constituent after 24 h. ERT = ertapenem, GC = growth control, KPC = *Klebsiella pneumoniae* carbapenemase, MEM = meropenem, MIC = minimal inhibitory concentration, TMP/SMX = trimethoprim/sulfamethoxazole.

However, on postoperative day 22 the patient became again febrile (T 39.0°C). At the physical examination, the patient was in poor condition, obtunded. White blood cells were 6820 cells/mmc (reference range 4000–10,000 cells/mmc; neutrophils, N 86%, reference range 40–70%), blood urea nitrogen (BUN) 52.7 mg/dL (reference range 12–25 mg/dL), creatinine 1.4 mg/dL (reference range 0.4–1.0 mg/dL), C-reactive protein (CRP) 84,000 μg/L (reference range 0–6000 μg/L), and erythrocyte sedimentation rate (ESR) 72 mm/h (reference range 0–20 mm/h). A chest X-ray showed a new consolidation in the left pulmonary lobe. Purulent discharge was present at the site of the recent abdominal drainage placement. Urine culture was sterile whereas blood (n = 2), BAL, and abdominal drainage cultures grew carbapenem-resistant (CR) *E coli* (Figure 1A). Thus, a diagnosis of surgical site infection and nosocomial pneumonia with concomitant bacteremia due to an MDR *E coli* was made. Despite the strain was sensitive to both aminoglycosides and colistin, the patient was considered to be at high risk of antibiotic-induced nephrotoxicity. Thus, in accordance to creatinine clearance (32 mL/min), antimicrobial treatment with ERT 500 mg every 24 h (1-h infusion) followed by high dose of MEM (2 g every 12 h, 3-h infusion) was started, in the absence of adverse effects. Considering that the administration of the double-carbapenem regimen has not been included in the clinical recommendation so far, the patient gave informed written consent for this unconventional therapeutic approach; thus, ethical approval was not necessary.

**FIGURE 2:**
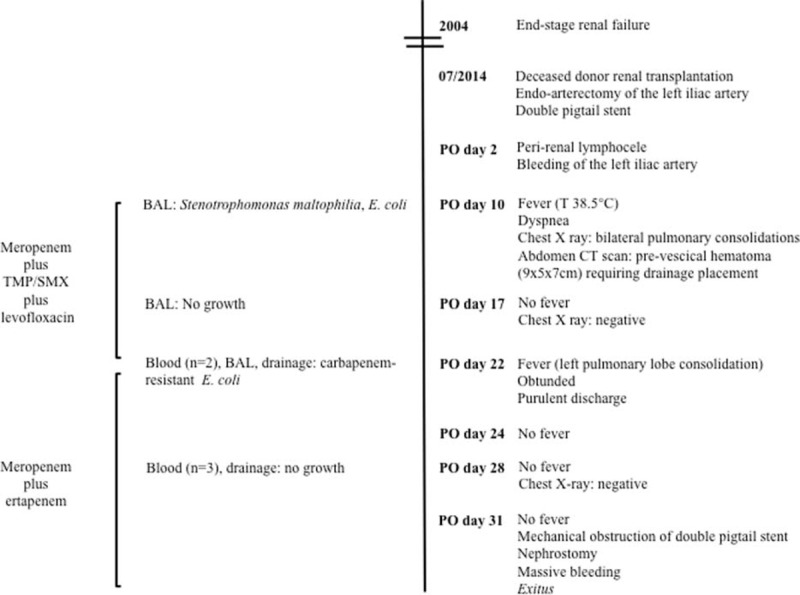
Timeline of clinical condition, interventions, and outcome. BAL = bronchoalveolar lavage, TMP/SMX = trimethoprim/ sulfamethoxazole, PO = postoperative.

After 96 h of such therapy, the patient became afebrile and the general conditions improved. Blood (n = 3) and drainage cultures were sterile. However, a mechanical obstruction of the double pigtail stent and a leakage at the level of anastomosis between bladder and left ureter were then observed. Creatinine increased to 2.4 mg/dL, BUN was 78.6 mg/dL, and a percutaneous nephrostomy was required. Three days later the patient suddenly worsened, blood pressure was 70/40 mm Hg, heart rate (HR) 130 per min, respiratory rate 35 beats/min, red blood cells 2,900,000 cells/mmc (reference range 4,000,000–5,400,000 cells/mmc), and hemoglobin 6.5 g/dL (reference range 11–14 g/dL). A radiological examination showed a massive bleeding at the level of surgical anastomosis. Despite a prompt vasopressor and inotropic support, the patient died (Figure 2).

Minimal inhibitory concentrations (MICs) of ERT and MEM were determined by broth macrodilution method (BMD) in cation-adjusted Mueller–Hinton broth.^12^

Diagnostic disks were used for the phenotypic determination of carbapenemases.^13^ For the molecular analysis, DNA was extracted from 1 colony of a fresh culture and then eluted in 100 μL of elution buffer (Qiagen, Milan, Italy). For amplification, the lyophilized PCR mix STATNAT DNA-Mix (Sentinel Diagnostics Robert Koch, Milan, Italy) containing 3.0 mM MgCl_2_, 0.8 mM each deoxynucleotide (dNTP), 2 U of hot-start *Taq* DNA polymerase, and reaction buffer was used. The mix was resuspended with 1 μL of the primer and probe mix and 1 μL of extracted DNA, with a final volume of 20 μL. The amplification was performed using a CFX 96 Real-Time System (Bio-Rad Laboratories, Marnes La Coquette, France).^14^

Furthermore, the activity of MEM and ERT, alone and in combination, was investigated by time-kill studies using an initial inoculum of ∼5 × 10^5^ CFU/mL. At 2, 4, 6, 8 and 24 h time points, the number of CFU was counted. The following concentrations were used: 1× MIC ERT, 1× MIC MEM, 2× MIC MEM, 0.5× MIC ERT + 0.5× MIC MEM, 1× MIC ERT + 0.5× MIC MEM, 1× MIC ERT + 1× MIC MEM, and 1× MIC ERT + 2× MIC MEM. Bactericidal activity was defined as a ≥3-log10 CFU/mL reduction of the initial bacterial count at each time point whereas synergy was defined as a ≥2-log10 decrease in CFU/mL between the combinations and its most active constituent after 24 h.

*E coli* MICs were 16 and 32 μg/mL for ERT and MEM, respectively. Phenotypic and molecular analyses showed that the strain was a KPC-producer.

In the killing curves, despite an initial reduction in log CFU/mL a regrowth at 24 h was observed for 1× MIC MEM and, to a lesser extent, for 1× MIC ERT; in contrast, 2× MIC MEM showed an absence of growth at 24 h. When the double-carbapenem combination was tested at concentrations of 1× MIC MEM + 1× MIC ERT, 0.5× MIC MEM + 1× MIC ERT, and 2× MIC MEM + 1× MIC ERT, a bactericidal activity was achieved at 4, 6, and 8 h and maintained up to 24 h with an absence of bacterial growth (Figure 1B).

## DISCUSSION

To our knowledge, this is the first report concerning a BSI caused by a KPC-producer *E coli* in a renal transplant patient treated with the double-carbapenem regimen, whose effectiveness was demonstrated throughout in vitro analyses.

In the recent years, the spread of CRE has become of major concern given that they show high levels of resistance to antimicrobial classes other than carbapenems.^2^ Although several mechanisms can lead to carbapenem resistance,^3,15^ much of the increase in CRE has been caused by the spread of carbapenemase-producing *K pneumoniae*, whereas the recent epidemiological data regarding the emergence of severe infections caused by KPC-producing *E coli*^16,17^ suggested that horizontal transfer of blaKPC genes among *Enterobacteriaceae* colonizing the human intestine may occur.

In transplant recipients, the impact of infections caused by MDR gram-negative bacteria, which generally occur in the first postoperative month,^11^ is a matter of concern because of the high mortality rates.^18^ Subjects could become colonized or infected with MDR microorganisms before or after transplantation.^19^

Furthermore, therapeutic options are worryingly limited since antimicrobials might exhibit toxicity and eventually interact with immunosuppressive agents.^11^ In this challenging setting, which seems to be even more complex due to the growing rate of resistance to colistin in CRE,^2^ innovative approaches including the double-carbapenem regimen have been proposed as a valid therapeutic option in severe infections due to CR *K pneumoniae*.^6–9^

In the present case, the strain showed in vitro sensitivity to both colistin and aminoglycosides. However, although in the presence of severe infections caused by resistant strains colistin-induced nephrotoxicity does not represent a major concern with a new formula of this drug, with regular monitoring of renal function, and with adequate kidney-based dose adjustment and hydration, the administration of these drugs was discouraged because of their well-known potential nephrotoxicity, especially if used together. Thus, based on the recent reports concerning the use of the double-carbapenem regimen,^6,20^ the patient was regarded as eligible for this unconventional therapeutic combination.

Despite high MICs to both ERT and MEM, our in vitro study demonstrated that the combination ERT plus MEM showed synergistic and bactericidal activity against a KPC-producing *E coli*. In particular, the combination 0.5 × MIC MEM + 1 × MIC ERT resulted to be highly bactericidal (Figure 1B), suggesting that even subinhibitory concentrations of MEM (16 μg/mL, which could be achieved in the serum after high dose and prolonged infusion of MEM) could be sufficient in order to exert its antibacterial activity.

The rationale of using the double-carbapenem regimen is based on the high affinity of carbapenemases for ERT, which binds to the hydrolytic enzymes and allows the other carbapenem to be effective.^20^ Furthermore, recent data support the hypothesis that high dose of carbapenems might reach adequate serum concentrations to achieve their pharmacokinetic target even against bacteria producing carbapenemases.^21^ Although higher concentration of MEM alone (2 × MIC, 64 μg/mL) had in vitro bactericidal effect similar to that of 0.5 × MIC MEM + 1 × MIC ERT (Figure 1B), we could speculate that, after high doses and prolonged infusion of MEM, the MEM concentrations achievable in the serum of patients are more likely closer to 16 μg/mL than to 64 μg/mL,^21^ which correspond to 0.5 × MIC MEM and 2 × MIC MEM in vitro concentrations, respectively.

In the present case, the double-carbapenem regimen was considered effective in view of both clinical and microbiological early responses (defervescence and negativity of blood and drainage cultures at 96 h, respectively). However, the patient experienced a worse outcome due to a massive bleeding at the level of surgical anastomosis.

The present report has some limitations. First, the inclusion of MEM in the treatment of the infection caused by *S maltophilia* and a pan-sensitive *E coli* might have contributed to the subsequent development of carbapenem resistance. Furthermore, although clinical outcomes with this regimen were favorable resulting in a rapid clearance of bacteremia, the patient died 9 days after the diagnosis for causes other than uncontrolled infection. Therefore, assessment of medium-term outcome and final definitive cure of the infection could not be allowed. Another limitation could have been obtaining synergistic antimicrobial in relation with the susceptibility profile of MEM and ERT, regarding the possibility of using carbapenems in cases with MIC values equal to or >32 μg/mL. However, throughout in vitro killing studies we were able to show how the combination 0.5 × MIC MEM + 1 × MIC ERT was bactericidal, supporting the hypothesis that ERT might have a prominent role in the combination by binding to carbapenemases and thus leading achievable serum concentration of MEM (ie, 16 μg/mL) to be effective.^6^

In conclusion, the present study showed the effectiveness of the double-carbapenem regimen in a transplant recipient with BSI due to CR *E coli*. This unconventional approach might be considered as a rescue therapy in those subjects, including transplant recipients, in whom previous antimicrobial combinations failed or when colistin use might be discouraged. Furthermore, this case outlines that performing in vitro synergy testing represents a useful strategy in order to select the best antimicrobial combinations, especially in cases of infections occurring in high-risk individuals such as transplant recipients.
